# A new forum for research on research integrity and peer review

**DOI:** 10.1186/s41073-016-0010-y

**Published:** 2016-05-03

**Authors:** Stephanie L. Harriman, Maria K. Kowalczuk, Iveta Simera, Elizabeth Wager

**Affiliations:** 1 BioMed Central, London, UK; 2 Centre for Statistics in Medicine, University of Oxford, Oxford, UK; 3 Sideview, Princes Risborough, Buckinghamshire, UK

## Abstract

This editorial explains why we are launching *Research Integrity and Peer Review*, a new open-access journal that will provide a home to research on ethics, reporting, and evaluation of research. We discuss how the idea to launch this journal came about and identify the gaps in knowledge where we would like to encourage more research and interdisciplinary discussion. We are particularly keen to receive submissions presenting actual research that will increase our understanding and suggest potential solutions to issues related to peer review, study reporting, and research and publication ethics.

## Main text

Welcome to the first edition of *Research Integrity and Peer Review*, a fully open-access journal considering submissions on all aspects of integrity in the research and publication process, including peer review, reporting, and research and publication ethics. We are excited to launch this new journal, which aims to provide a dedicated forum for publishing research into this increasingly recognized field.

Academic research affects the lives of everybody who has ever used a car, a phone, or a computer, or who has ever taken a medicine. Research also influences the way we educate our children, organize our hospitals, and punish our criminals. If that research is unreliable, we may waste money and even lives. And if that research is not communicated effectively, its conclusions may be distorted or its messages ignored [1]. For research to do good rather than harm, it must have integrity and it must be reported properly. But research is a complex activity, which may be described (using a suitably complex term) as a bio-psychosocial function—or, put more simply, prone to human fallibilities. Getting research and its communication “right” is not always as simple as it sounds.

To reap the benefits of research, we need to understand it properly. We need to consider questions such as what causes research to give misleading results, what tempts researchers to cheat, and how best to report and disseminate research findings. In other words, we need research into research, and we need somewhere to publish these findings.

Although academic enquiry—and reports of it—have existed for centuries, serious study of research is a surprisingly recent phenomenon. About 25 years ago, Drummond Rennie, a visionary editor at *JAMA* (the *Journal of the American Medical Association*) wrote that “the vast majority of papers written about editorial peer review had been composed in the absence of any data and were editorial effusions that expressed individual biases” [2]. He therefore established the international peer review congresses, starting in 1989, the eighth of which is due to take place in September 2017. Since the first congress, research into peer review (both of journals and for funding) has increased and evolved [3].

It is a similar story for the study of research reporting. Although randomized clinical trials as we know them today have been published since the 1940s [4], the first major reporting guideline in medical research, CONSORT (the Consolidated Statement on Reporting Trials) was only published in 1996 [5].

Since then, many other reporting guidelines have been published to guide the reporting of various study designs in a range of disciplines [6]. However, despite this pioneering work, the reporting of clinical trials, which forms the backbone of the medical evidence base, remains problematic [7]. We lack research on how best to implement reporting guidelines and other tools to support researchers and reviewers in achieving high standards in research reporting. We lack research proposing and evaluating changes in reward and research systems that aim to maximize the value of research.

The journal’s third theme of research integrity has also become increasingly recognized in the last decade, having had its own international conference, the World Conference on Research Integrity, since 2007. The term “research integrity” describes a situation where research is conducted and reported in such a way that it produces useful, trustworthy results and encompasses the entire process from the planning of research right through to the dissemination of research findings. It affects (and is the responsibility of) all those involved in the process.

While the growing interest in research integrity, reporting, and publication is encouraging, huge gaps in our knowledge remain. Until now, the little research that has been done has been scattered across the literature, often inaccessible to those without subscriptions to journals. Another problem has been that research that might be applicable across a number of disciplines has remained invisible to many researchers and editors who might benefit from it, because it was published only in the journals where the research was done, so people working in other fields were unaware of it (evolutionary biologists do not generally look at anesthesiology journals, for example).

Our aims in launching *Research Integrity and Peer Review* are to bring this work together and stimulate interdisciplinary discussion, ensuring that all those who can benefit from these research findings have access to them. Further to these aims, the journal is fully online and open access, ensuring that such research is fully available to everyone who may benefit from it.

We look forward to receiving submissions on research and publication ethics, research reporting, and peer review. These topics will be handled by the four co-Editors-in-Chief, Maria Kowalczuk, Stephanie Harriman, Iveta Simera, and Elizabeth Wager, respectively. Like Drummond Rennie, we are less interested in “editorial effusions” but more interested in research that illuminates any aspect of these topics. The journal has a particular focus on submissions that present research and those that offer potential solutions to current controversies and limitations in the field.

While much is written about the problems and limitations, particularly of peer review, this is often in the form of blogs and opinion pieces and does not offer empirical evidence or solutions. Under the heading of research, we include not only formal, funded projects but also smaller scale initiatives or audits undertaken by journals or funders to monitor and improve their processes.

Given the lack of research and therefore of understanding about peer review, we aim to be open to experiment in this area. To ensure transparency in the peer review process, the journal will operate on an open peer review model, building on practices pioneered by BioMed Central since 2000. This involves two levels of openness: authors are aware of the identity of the peer reviewers and, if the article is published, the reviewers’ reports are available to readers alongside the published article.

## Conclusions

We hope you share our excitement at the launch of this new journal and we welcome—and thank in anticipation—our readers, peer reviewers, authors, and editorial board members. The success of this journal lies with us all—please join us for what we hope will be a fascinating and rewarding journey.
